# Impact of Insulin Resistance on Ovarian Sensitivity and Pregnancy Outcomes in Patients with Polycystic Ovary Syndrome Undergoing IVF

**DOI:** 10.3390/jcm12030818

**Published:** 2023-01-19

**Authors:** Zhuoye Luo, Lili Wang, Yizhuo Wang, Yanli Fan, Lei Jiang, Xin Xu, Yuanjie Du, Guimin Hao

**Affiliations:** Hebei Clinical Research Center for Birth Defects, Department of Reproductive Medicine, The Second Hospital of Hebei Medical University, Shijiazhuang 050000, China

**Keywords:** in vitro fertilization, insulin resistance, ovarian sensitivity index, polycystic ovary syndrome, early miscarriage rate

## Abstract

Background: Ovarian sensitivity index (OSI) is an accurate index to reflect the ovarian sensitivity to exogenous gonadotropins in in vitro fertilization (IVF). How insulin resistance (IR) affects OSI and pregnancy outcomes during IVF remains unclear. Methods: This was a large retrospective, cohort study. A total of 2055 women with polycystic ovary syndrome (PCOS) undergoing the first fresh IVF cycle were enrolled. They were grouped into terciles based on the homeostasis model assessment of insulin resistance (HOMA-IR) values as control, medium and IR group for comparison. Multivariate regression analysis was also conducted. Results: HOMA-IR had a significantly negative impact on OSI (adjusted β = −0.24; 95% CI, −0.35 to −0.13), especially in lean patients with an adjusted β of −0.33 (95% CI, −0.51 to −0.16). The interaction analysis revealed an interactive association between HOMA-IR and body mass index (BMI) (*p* = 0.017). IR was related to an increased early miscarriage risk independently with an odds ratio (OR) of 2.21 (95% CI, 1.13 to 4.33), without significant impact on pregnancy and live birth rate. Conclusion: IR decreased the ovarian response in PCOS patients undergoing IVF, especially in the lean subgroup. IR may result in a higher risk of early miscarriage, but did not impair pregnancy and live birth rate.

## 1. Introduction

Polycystic ovary syndrome (PCOS) is a prevalent multi-phenotypic and complex endocrine and metabolic disease, covering 6% to 20% women of reproductive age worldwide based on racial diversity and various diagnostic criteria [1,2]. The crucial pathophysiological features of PCOS include insulin resistance (IR), hyperinsulinemia, and hyperandrogenemia, which leads to clinical features such as oligomenorrhea, anovulation, polycystic ovary morphology, hirsutism, and acne [3]. 

Approximately 50–70% of PCOS patients have insulin resistance, a pathological status in which the capacity of insulin to regulate glucose metabolism is impaired [4]. The “gold standard” for evaluating IR is the euglycemic-hyperinsulinemic clamp test [5], which is usually only used in research as it is complex and invasive. Instead, the homeostasis model assessment of insulin resistance (HOMA-IR) is commonly used in clinical settings, but its cutoff suffers from a great lack of uniformity [6]. 

Insulin functions physiologically as a co-gonadotropin to regulate follicle growth and development [7]. In the human ovary, insulin receptors have been discovered to be located on multiple types of cells, such as stromal, granulosa, and theca cells [8,9]. Insulin also amplifies the effects of insulin-like growth factor-I (IGF-I) by upregulating the expression of ovarian IGF-I receptors. However, IR and hyperinsulinemia may insult follicle development and cause reproductive disorders in PCOS [10]. 

Patients with PCOS are more likely to seek in vitro fertilization (IVF) for pregnancy. The ovarian response to exogenous gonadotropin has an obvious effect on the efficacy of IVF treatment. Conflicting conclusions were reached in previous studies about how IR affected ovarian response and pregnancy outcomes [11,12,13,14,15]. Some of these studies were less convincing for small sample sizes or non-standardized stimulation protocols. Furthermore, these studies employed a dominant follicle count or the number of oocytes retrieved as indicators for ovarian response. These indicators were dependent on the exogenous gonadotropin dosage, making them lack accuracy. Artificial error was indispensable when measuring follicle count [16,17,18,19].

A better indicator was needed to evaluate ovarian response. Ovarian sensitivity index (OSI), an index to indicate the exogenous gonadotropins dosage required for per oocyte retrieved, is an accurate parameter to reflect the ovarian sensitivity to gonadotropins as well as predict pregnancy outcome [20,21]. OSI also has good inter-cycle consistency, providing better guidance for tailored, individualized treatment in subsequent cycles [20,22]. However, previous studies rarely focused on the relationship between IR and OSI, so it remains unclear.

In this large-scale study, we evaluated the effect of IR on OSI-reflected ovarian response and IVF outcomes among PCOS patients. Our findings aim to provide evidence for individualized ovarian stimulation, highlighting early evaluation and interventions of IR before IVF to improve pregnancy outcomes.

## 2. Materials and Methods

### 2.1. Study Design and Patients

A retrospective study was performed at our hospital from March 2017 to April 2022 following the Strengthening the Reporting of Observational Studies in Epidemiology (STROBE) guidelines. This study was approved by the ethics committee of the Second Hospital of Hebei Medical University (No. 2017-R004). There is no requirement for informed consent. 

The inclusion criteria were as follows: (1) aged between 20–40 years with basal follicle-stimulating hormone (FSH) less than 12 IU/L, (2) diagnosed PCOS according to Rotterdam criteria [23,24], and PCOS was the only infertile factor besides possible tubal factors, (3) received gonadotropin-releasing hormone (GnRH) antagonist or GnRH agonist-short protocol for the first fresh cycle of IVF. Patients with uterine malformations or endocrine diseases (i.e., thyroid dysfunction, hyperprolactinemia and type II diabetes mellitus), or who underwent preimplantation genetic testing, or took drugs that could affect metabolism or plasma steroid levels within 3 months before the study, were excluded. Patients were also excluded if they missed important information (i.e., information about fasting, insulin or glucose concentrations, or pregnancy outcomes). Finally, a total of 2055 patients were enrolled. 

Since there are no consensus HOMA-IR cutoffs to diagnose IR, patients were grouped into terciles according to their HOMA-IR values for comparison. For sensitivity analysis, we also set the cutoff at 2.56 based on the highest quartiles of HOMA-IR value in lean (body mass index < 25 kg/m^2^) non-PCOS control in our center [25].

### 2.2. Controlled Ovarian Stimulation Protocol

The patients received GnRH antagonist or GnRH agonist-short protocol. Recombinant follicle-stimulating hormone (rFSH) (Gonal-F; Merck Serono, Pharma S. p.A., Bari, Italy) was initially administrated during the 2nd to 3rd day of the menstrual cycle or after downregulation for 14–16 days. Follicular growth was monitored by transvaginal ultrasound. 250 μg of GnRH antagonist (Cetrorelix; Merck Serono, Pharma S. p.A., Bari, Italy) was used daily after the diameter of leading follicle reached 12 mm. Between 6500–10,000 IU of human chorionic gonadotrophin (hCG) was injected to induce ovulation when two or more leading follicles exceeded 18 mm. Oocytes retrieval was started 36–38 h later and then inseminated by IVF or intracytoplasmic sperm injections (ICSI) based on infertility reason. Fresh embryo was transferred between 3–5 days following oocyte retrieval. Progesterone gel (Crinone, Merck Serono, Watford, UK) was started for luteal phase support on the day of oocyte pick-up, lasting to 8 weeks of gestation if conception occurs.

### 2.3. Hormone Measurements

All measurement were conducted using the Immunoassay System of Immulite 2000 (Siemens Healthcare Global, Munich, Germany). Basal hormones (including basal FSH, luteinizing hormone (LH), testosterone (T) and anti-Müllerian hormone (AMH)) were collected during the 2nd or 3rd day of the menstrual cycle before medication. Insulin, glucose, total cholesterol and triglyceride concentrations were collected between 7:30 and 9:30 after overnight fasting. FSH, LH and estradiol (E_2_) were monitored during ovarian stimulation.

### 2.4. Indicators

Body mass index (BMI) was calculated as body weight (kg)/height (m^2^). HOMA-IR was calculated as fasting glucose (mmol/L) × fasting insulin (μIU/mL)/22.5. OSI was calculated as the number of retrieved oocytes/total gonadotropin dose (IU) × 1000. 

Biochemical pregnancy was diagnosed when serum hCG level reached more than 5 IU/L between 12–14 days following embryo transfer. Clinical pregnancy was confirmed by visuals of an intrauterine gestational sac with transvaginal ultrasound 4–5 weeks after embryo transfer. Miscarriage was defined as suffering pregnancy loss before 28 weeks gestation after achieving clinical pregnancy. Early miscarriage was defined as suffering from miscarriage less than 12 gestational weeks. Live birth was defined as delivering at least one living child.

### 2.5. Statistical Analysis

Patients were grouped into terciles according to their HOMA-IR values. Continuous variables were expressed as mean ± standard deviation (mean ± SD), with One-way ANOVA or Kruskal Wallis test for comparison. Pearson’s chi-square analysis was applied in categorical variables. Linear regression and spline fitting was used to evaluate the relationship between HOMA-IR and OSI. Test for interaction was performed according to BMI subgroups. Logistic regression analysis was conducted to manifest how IR affected pregnancy outcomes. For sensitivity analysis, we set the HOMA-IR cutoff at 2.56 and considered it an exposure factor to perform multivariable regression analysis. Two models were built for multivariable regression, with age and BMI treated as categorical variables in model I and as continuous variables in model II. Adjusted confounders were chosen based on a 10% change in the effect estimate. All statistical analyses were carried out with the statistical package R version 4.2.1 (http://www.R-project.org, accessed on 1 November 2022, R Foundation for Statistical Computing, Vienna, Austria) and EmpowerStats (http://www.empowerstats.com, accessed on 1 November 2022, X&Y Solution, Inc., Boston, MA, USA) [26,27,28]. A two-sided *p*-value < 0.05 was statistically significant.

## 3. Results

### 3.1. Demographic and Characteristics

**Figure 1:**
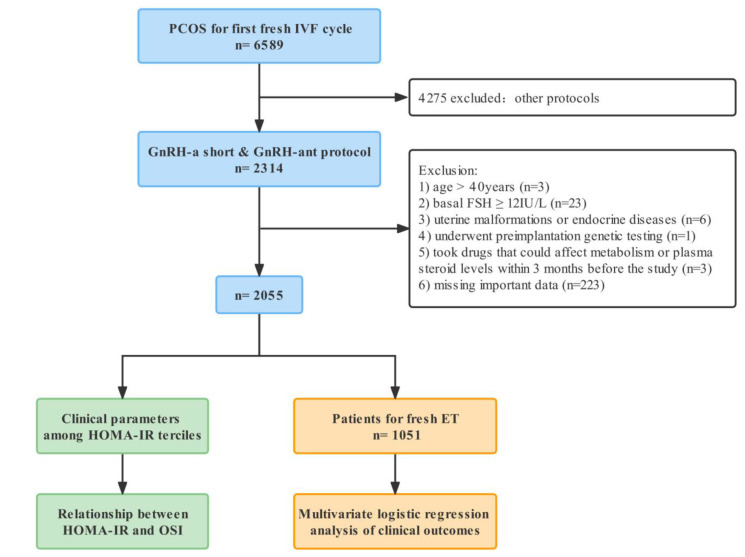
Flow chart for selection. PCOS, polycystic ovary syndrome; IVF, in vitro fertilization; GnRH-a, gonadotropin-releasing hormone agonist; GnRH-ant, gonadotropin-releasing hormone antagonist; FSH, follicle-stimulating hormone; HOMA-IR, homeostatic model assessment of insulin resistance; OSI, ovarian sensitivity index; ET, embryo transfer.

A total of 2055 patients were enrolled in this study (Figure 1). Patients were grouped into terciles based on their HOMA-IR values, and the cutoffs were found to be 1.97 and 3.71. The demographics and characteristics of patients among three groups are presented in Table 1. It is obvious that those in the higher HOMA-IR groups had significantly greater fasting glucose and fasting insulin. BMI, blood pressure, triglyceride and cholesterol significantly increased with HOMA-IR. The basal LH and basal T were significantly increased with higher HOMA-IR. While patients in the three groups were of similar age, the serum basal FSH and AMH were significantly lower with HOMA-IR increased. Patients with higher HOMA-IR required a significantly higher FSH initial dosage, total dosage and days of stimulation in spite of fewer down-regulation protocol being conducted. E_2_ on trigger day, the number of oocytes retrieved and OSI were all decreased significantly with higher HOMA-IR. PCOS can be classified into four phenotypes according to different combination of symptoms [23,24]: hyperandrogenism (HA) + oligo-anovulation (OA) + polycystic ovaries (defined by ultrasound) (PCO), HA + OA, HA + PCO and OA + PCO. Infertility duration, infertility type, antral follicles count (AFC) and PCOS phenotypes were of no significant difference among the groups.

### 3.2. Relationship between Clinical Characteristics and OSI

Univariate linear regression analysis of clinical characteristics to OSI were shown in Table 2. The coefficient β represented the change of OSI caused by per one-unit change of a specific variable. Age, infertility duration and basal FSH had significantly negative impacts on OSI, while AMH was opposite. BMI, triglyceride, fasting glucose, fasting insulin and HOMA-IR had significantly negative impacts on OSI. PCOS phenotypes and variables related (basal LH, basal T and AFC) had significantly positive or negative impacts on OSI. Patients who needed a higher FSH initial dosage, longer duration of stimulation or with lower E_2_ on trigger day showed significantly decreased OSI. Infertility type, blood pressure and cholesterol had no significant effect on OSI (*p* > 0.05).

### 3.3. Effect of HOMA-IR on OSI in All Patients and BMI Subgroups

**Figure 2:**
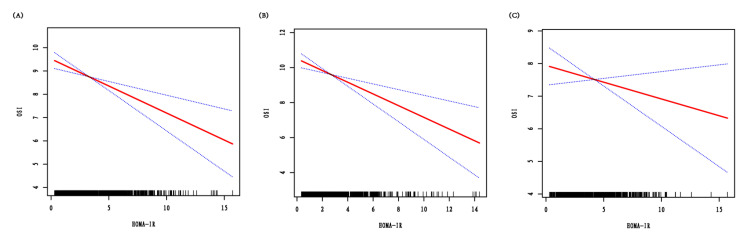
Multivariate adjusted smoothing spline plots of OSI by HOMA-IR. (**A**) for all PCOS. (**B**) for lean (BMI < 25 kg/m^2^) PCOS. (**C**) for overweight/obese (BMI ≥ 25 kg/m^2^) PCOS. Solid lines demonstrate the relationship between HOMA-IR and OSI. Dotted curves or shaded areas represent the 95% of confidence intervals. Adjusted for: age subgroups; BMI subgroups; infertility time; AMH; basal FSH; basal LH; basal T; AFC; PCOS phenotypes; triglyceride; protocol. HOMA-IR, homeostatic model assessment of insulin resistance; OSI, ovarian sensitivity index; BMI, body mass index; AMH, anti-Müllerian hormone; FSH, follicle-stimulating hormone; LH, luteinizing hormone; T, testosterone; AFC, antral follicle count; PCOS, polycystic ovary syndrome.

Multivariate linear regression was conducted to investigate how HOMA-IR affected OSI. Variables adjusted were as follows: age subgroups, BMI subgroups, infertility time, AMH, basal FSH, basal LH, basal T, AFC, PCOS phenotypes, triglyceride and protocol. HOMA-IR terciles and HOMA-IR category based on cutoff of our center was also analyzed as sensitivity analysis. After adjusting for confounding variables, HOMA-IR still had a significantly negative impact of on OSI (adjusted β = −0.24; 95% CI, −0.35 to −0.13), especially in lean patients with an adjusted β of −0.33 (95% CI, −0.51 to −0.16). The interaction analysis revealed an interactive association between HOMA-IR and BMI (*p* = 0.017) (Table 3). Smooth curve fitting showed a linear relationship of HOMA-IR with OSI (Figure 2). All results were tested similarly in model II (Supplementary Table S1). 

### 3.4. Effect of HOMA-IR on Pregnancy Outcomes

Multivariate logistic regression was conducted to verify how HOMA-IR affected pregnancy outcomes. Patients who transferred their embryos in the fresh cycle were included in this part. Two regression models were built according to clinical experience and univariate analysis results (Table 4). IR defined by HOMA-IR ≥ 2.56 played an independent role in early miscarriages in both models. The group 3 of HOMA-IR terciles had a significantly higher early miscarriage rate than that of group 1. HOMA-IR had no significant effect on other pregnancy outcomes.

## 4. Discussion

Our study indicated that IR had a significantly negative effect on ovarian response in PCOS patients who had conducted IVF, with a greater influence on the lean subgroup compared to their overweight/obese counterparts. In addition, patients with IR presented a higher risk of early miscarriage.

### 4.1. The Cutoffs of HOMA-IR

As mentioned above, there are no consensus HOMA-IR cutoffs to diagnose IR. For the convenience to compare, patients were grouped into terciles based on their HOMA-IR values, called group 1 to 3, with the cutoffs being 1.97 and 3.71. We also tried to find a proper cutoff for sensitivity analysis. Different cutoffs were set in previous authoritative studies, varying from 2.2 to 2.77 based on diverse populations [25,29,30,31]. Since many clinical cutoffs were set according to the 75th or 95th percentiles value in common population, it is more convincing to set the 75th or 95th percentiles of HOMA-IR values in lean (body mass index < 25 kg/m^2^) non-PCOS control population as the cutoff to diagnosis IR [6]. This cutoff was 2.56 in our center, which was just in the range of 2.2–2.77. As a result, we set the cutoff to IR diagnosis at 2.56 as the second classification method in our study for sensitivity analysis. Therefore, group 1 can be considered as the control group and group 3 were classified as the IR group, with group 2 being a medium status. We found the results between the two classification methods were in accord with each other.

### 4.2. Clinical Characteristics and IR

PCOS shows a great resemblance to metabolic syndrome, which combines abdominal obesity, IR, dyslipidemia and hypertension [13]. It is reasonable to find that BMI, blood pressure, triglyceride and cholesterol significantly increased with HOMA-IR. Our results were consistent with those of previous studies [32,33]. Additionally, we demonstrated that basal LH and T were significantly higher in groups of higher HOMA-IR, which was also in line with another study [34]. Excessive insulin may promote T production by facilitating P450 c17α enzyme synthesis, as well as upregulating LH receptor of theca cells [35,36]. Excessive insulin also increased the proportion of free T, an active status for T to play a biological role, by suppressing the hepatic sex hormone-binding globulin (SHBG) synthesis and reducing circulated SHBG [37,38]. Hyperinsulinemia may aggravate hyperandrogenism, and androgen excess, in turn, may induce IR and compensatory hyperinsulinemia, further formulating a vicious circle in patients with PCOS. 

### 4.3. IR Affecting Ovarian Response

The ovarian response to exogenous FSH is critical to ovarian stimulation during IVF. It has remained unclear how IR affects ovarian response. We found that patients in the higher HOMA-IR group required a significantly higher FSH dosage as well as a longer duration of stimulation. They presented significantly lower E_2_ on trigger day with fewer oocyte number retrieved, which suggested that PCOS patients with IR may be less sensitive to FSH. Our findings agreed with the study reported by Hassani et al. [11]. A similar result was observed in PCOS patients with metabolic syndrome [13]. Fedorcsák et al. discovered that metformin treatment for 1500 mg/d increased the number of oocytes retrieved [12], which suggested that IR compromised follicular recruitment. In vitro, Xu et al. found that E_2_ secretion was decreased from the granular cell line after treated by insulin [39], which was in line with our results in vivo. Anjali G et al. discovered that the FSH receptor (FSHR) presented a selective activate deficiency in PCOS patients [40], and the high LH receptor (LHR) further impaired glucose metabolic reaction to FSH in granulosa cells [41]. 

However, there also exists a study with an opposite opinion. Fleming et al. considered that follicles in PCOS patients with IR maintained sensitivity to exogenous FSH for a longer time before atresia, but the number of FSH-sensitive follicles didn’t increase. As a result, IR had limited effects on the number of oocytes retrieved [14]. Another study with a small sample size concluded that patients with hyperinsulinemia show better ovarian response [15]. These studies differed from our study obviously in protocols or methods defining IR, which may affect follicular development dynamics. That may partially explain the discrepant results. Insulin functions physiologically as a co-gonadotropin to regulate follicle growth and development [8,9], but too much insulin may downregulate the receptors’ expression and impair glucose uptake, having an adverse effect on follicles [42]. We speculated that hyperinsulinemia in follicles may impede the interaction between granulosa cells, restrain aromatase activity, retard the growth of follicles and finally decrease ovarian response.

OSI is considered as a reliable index to reflect the ovarian sensitivity to gonadotropins and to guide tailored, individualized administration in subsequent cycles [20,21]. Currently, only one study with a smaller sample size mentioned IR and OSI, however, the clinical parameters in each group were not matched [43]. Our study first confirmed that IR significantly decreased OSI in PCOS patients by multivariate regression. It is noted that a greater negative influence was found in lean PCOS patients compared to their overweight/obese counterparts, with HOMA-IR and BMI presenting an interactive association based on the interaction analysis. Our study reveals a unique finding unavailable in previous studies, supporting the theory that different mechanisms may lie in the formation of IR between lean and obese PCOS [44,45]. It is suggested that the initial FSH dosage should be increased properly to obtain the anticipated oocytes number in PCOS patients with serious IR, especially for lean ones. LH should also be paid attention to, to reach a certain level.

### 4.4. IR and Pregnancy Outcomes

Previous studies have shown that OSI play an independent role in predicting the pregnancy rate [21], but our results revealed that higher HOMA-IR did not reduce the pregnancy rate while OSI decreased. We found that OSI decreased by 0.24 as HOMA-IR increased per unit, which represented the number of oocytes retrieved that would decrease by 0.5 correspondingly, based on the mean total FSH of 2200 IU in our study. It has been demonstrated that the live birth rate was nearly similar when more than 10–15 oocytes were retrieved [46]. Fewer oocytes retrieved may have limited influence on the pregnancy rate in patients with PCOS who usually have a good ovarian reserve. Our study indicated that patients with IR presented a higher risk of early miscarriage, which was in line with the majority of previous studies [47,48,49]. It has been postulated that oxidative stress imbalance, mitochondrial abnormalities and gut microbiota dysbiosis may be involved in the cellular mechanism [50]. 

### 4.5. Strengths and Limitations

To our best knowledge, our study first confirmed that IR has a negative influence on ovarian response in patients with PCOS by multivariate regression analysis, and we had a novel finding that this influence was greater for lean ones. These may provide new ideas for future research. Secondly, a high consistency was reached in medication and hormone measurement within a single center. Lastly, sensitivity analysis, stratified analysis and interaction test were carried out on the basis of multivariate regression analysis with a large sample size, which has improved the reliability of our conclusion. However, our study has several limitations. As a retrospective study, bias was inevitable. Our results will be more convincing if similar results were concluded when another validated surrogate marker for IR was used, such as the quantitative insulin sensitivity check index (QUICKI) [51]. In addition, our results cannot be extrapolated to the non-PCOS population. Further research is required to confirm and extend our observations and to elucidate the mechanisms behind them.

## 5. Conclusions

In conclusion, our study showed that IR had a significantly adverse effect on the ovarian response in PCOS patients who conducted IVF, and we discovered greater influence on the lean subgroup compared to their overweight/obese counterparts for the first time. Furthermore, we confirmed that patients with IR presented a higher risk of early miscarriage. It is recommended that early evaluation and interventions of IR should be highlighted prior to IVF, especially for lean PCOS patients. Further studies are required to verify our observations and to find out the proper interventions to improve pregnancy outcomes.

## Figures and Tables

**Table 1:** The demographic and characteristics of patients among three groups.

Characteristics	HOMA-IR ≤ 1.97 (n = 685)	1.97 < HOMA-IR ≤ 3.71 (n = 685)	HOMA-IR > 3.71 (n = 685)	*p*-Value
Age (years)	29.12 ± 3.61	29.25 ± 3.64	29.28 ± 3.89	0.698
BMI (kg/m^2^)	22.48 ± 3.24	24.39 ± 3.44	26.20 ± 3.53	<0.001
Infertility duration (years)	3.49 ± 2.54	3.67 ± 2.28	3.77 ± 2.74	0.114
Infertility type				0.298
Primary infertility	475 (69.34%)	461 (67.30%)	448 (65.40%)	
Secondary infertility	210 (30.66%)	224 (32.70%)	237 (34.60%)	
AMH (ng/mL)	7.59 ± 3.39	7.13 ± 3.26	6.80 ± 2.98	<0.001
Basal FSH (IU/L)	7.00 ± 1.81	6.86 ± 1.75	6.75 ± 1.77	0.038
Basal LH (IU/L)	6.94 ± 4.95	7.24 ± 5.23	7.43 ± 4.85	0.016
Basal T (ng/mL)	0.61 ± 0.34	0.65 ± 0.36	0.66 ± 0.36	0.011 *
AFC	19.58 ± 7.02	19.54 ± 6.89	19.91 ± 6.93	0.556
PCOS phenotypes				0.105
HA + OA + PCO	130 (18.98%)	124 (18.10%)	118 (17.23%)	
HA + OA	93 (13.58%)	97 (14.16%)	89 (12.99%)	
HA + PCO	205 (29.93%)	206 (30.07%)	172 (25.11%)	
OA + PCO	257 (37.52%)	258 (37.66%)	306 (44.67%)	
Blood pressure				
Systolic (mmHg)	112.14 ± 8.04	113.07 ± 8.20	114.28 ± 10.23	<0.001
Diastolic (mmHg)	72.72 ± 6.68	73.49 ± 7.12	74.17 ± 7.94	0.001
Triglyceride (mmol/L)	1.11 ± 0.57	1.32 ± 0.60	1.48 ± 0.79	<0.001 *
Cholesterol (mmol/L)	4.37 ± 0.68	4.40 ± 0.66	4.49 ± 0.69	0.002
Fasting glucose (mmol/L)	5.16 ± 0.36	5.28 ± 0.36	5.46 ± 0.38	<0.001
Fasting insulin (mIU/L)	5.48 ± 2.03	11.54 ± 2.07	24.58 ± 7.22	<0.001
HOMA-IR	1.25 ± 0.46	2.70 ± 0.48	5.98 ± 1.89	<0.001
Protocol				<0.001
GnRH-antagonist	211 (30.80%)	256 (37.37%)	277 (40.44%)	
GnRH-antagonist	474 (69.20%)	429 (62.63%)	408 (59.56%)	
FSH initial dosage (IU)	189.21 ± 48.83	197.52 ± 49.94	205.55 ± 52.07	<0.001
FSH total dosage (IU)	2049.24 ± 747.39	2206.55 ± 849.45	2379.65 ± 926.39	<0.001
Duration of COS (days)	10.28 ± 2.69	10.36 ± 2.45	10.68 ± 2.58	0.012
E_2_ on trigger day (pg/mL)	4285.59 ± 1158.08	3982.59 ± 1379.20	3825.08 ± 1385.84	<0.001
No. of oocyte retrieved	17.28 ± 10.06	16.38 ± 9.11	15.13 ± 8.10	0.005 *
OSI	9.90 ± 7.57	8.88 ± 6.73	7.38 ± 4.83	<0.001 *

Data are presented as either means ± SD or number (%). Distributions were compared using One-way ANOVA or Kruskal Wallis test (*). Categorical variables were compared using Pearson’s chi-square test. BMI, body mass index; AMH, anti-Müllerian hormone; FSH, follicle-stimulating hormone; LH, luteinizing hormone; T, testosterone; AFC, antral follicle count; PCOS, polycystic ovary syndrome; HA, hyperandrogenism; OA, oligo-anovulation; PCO, polycystic ovaries (defined by ultrasound); HOMA-IR, homeostatic model assessment of insulin resistance; GnRH, gonadotropin-releasing hormone; COS, controlled ovarian stimulation; OSI, ovarian sensitivity index.

**Table 2:** Univariate linear regression analysis of some clinical characteristics to ovarian sensitivity index (OSI).

Variable	Value	Crude β (95% CI)	*p*-Value
Age (years)	29.22 ± 3.72	−0.26 (−0.33, −0.18)	<0.0001
Age subgroups (years)			
[20, 30)	1184 (57.62%)	0	
[30, 35)	686 (33.38%)	−0.53 (−0.94, 0.29)	0.297
[35, 40]	185 (9.00%)	−2.94 (−3.96, −1.93)	<0.0001
BMI (kg/m^2^)	24.35 ± 3.73	−0.31 (−0.39, −0.24)	<0.0001
BMI subgroups (kg/m^2^)			
<18.5	87 (4.26%)	0	
[18.5, 25)	1106 (54.11%)	−0.50 (−1.92, 0.91)	0.486
[25, 30)	701 (34.30%)	−2.31 (−3.76, −0.87)	0.0017
≥30	150 (7.34%)	−3.82 (−5.53, −2.10)	<0.0001
Infertility duration (years)	3.64 ± 2.53	−0.28 (−0.39, −0.17)	<0.0001
Infertility type			
Primary infertility	1384 (67.35%)	0	
Secondary infertility	671 (32.65%)	−0.34 (−0.95, 0.27)	0.272
AMH (ng/mL)	7.17 ± 3.23	0.94 (0.86, 1.01)	<0.0001
Basal FSH (IU/L)	6.87 ± 1.78	−0.94 (−1.10, −0.78)	<0.0001
Basal LH (IU/L)	7.21 ± 5.02	0.28 (0.22, 0.34)	<0.0001
Basal T (ng/mL)	0.94 ± 0.52	−2.31 (−2.85, −1.77)	<0.0001
AFC	19.68 ± 6.95	0.39 (0.36, 0.43)	<0.0001
PCOS phenotypes			
HA + OA + PCO	372 (18.10%)	0	
HA + OA	279 (13.58%)	−3.88 (−4.86, −2.89)	<0.0001
HA + PCO	583 (28.37%)	−3.71 (−4.54, −2.89)	<0.0001
OA + PCO	821 (39.95%)	−0.57 (−1.35, 0.21)	0.150
Blood pressure			
Systolic (mmHg)	113.16 ± 8.91	0.00 (−0.03, 0.04)	0.844
Diastolic (mmHg)	73.46 ± 7.29	−0.03 (−0.07, 0.01)	0.128
Triglyceride (mmol/L)	1.30 ± 0.68	−1.15 (−1.57, −0.73)	<0.0001
Cholesterol (mmol/L)	4.42 ± 0.67	0.03 (−0.39, 0.45)	0.882
Fasting glucose (mmol/L)	5.30 ± 0.39	−1.50 (−2.23, −0.77)	<0.0001
Fasting insulin (mIU/L)	13.87 ± 9.15	−0.12 (−0.15, −0.09)	<0.0001
HOMA-IR	3.31 ± 2.29	−0.50 (−0.62, −0.37)	<0.0001
HOMA-IR Tercile			
Low	685 (33.33%)	0	
Middle	685 (33.33%)	−1.03 (−1.71, −0.34)	0.0035
High	685 (33.33%)	−2.52 (−3.21, −1.84)	<0.0001
Protocol			
GnRH-antagonist	744 (36.20%)	0	
GnRH-antagonist	1311 (63.80%)	−0.81 (−1.40, −0.22)	0.0075
FSH initial dosage (IU)	197.44 ± 50.71	−0.06 (−0.06, −0.05)	<0.0001
Duration of COS (days)	10.44 ± 2.58	−0.69 (−0.80, −0.58)	<0.0001
E_2_ on trigger day (pg/mL)	4032.31 ± 1324.82	0.00 (0.00, 0.00) *	<0.0001

Data are presented as either means ± SD or number (%). BMI, body mass index; AMH, anti-Müllerian hormone; FSH, follicle-stimulating hormone; LH, luteinizing hormone; T, testosterone; AFC, antral follicle count; PCOS, polycystic ovary syndrome; HA, hyperandrogenism; OA, oligo-anovulation; PCO, polycystic ovaries (defined by ultrasound); HOMA-IR, homeostatic model assessment of insulin resistance; GnRH, gonadotropin-releasing hormone; COS, controlled ovarian stimulation. * With three digits after the decimal point: 0.003(0.002, 0.003).

**Table 3:** Multivariate linear regression analysis of HOMA-IR to ovarian sensitivity index (OSI) in all patients and BMI subgroups.

Variable	All Patients		Lean (BMI < 25 kg/m^2^)		Overweight to Obese (BMI ≥ 25 kg/m^2^)		Test for Interaction
	Adjusted β (95% CI)	*p*-Value	Adjusted β (95% CI)	*p*-Value	Adjusted β (95% CI)	*p*-Value	*p*-Value
HOMA-IR	−0.24 (−0.35, −0.13)	<0.0001	−0.33 (−0.51, −0.16)	<0.0001	−0.12 (−0.27, 0.02)	0.092	0.017
HOMA-IR Tercile							0.212
Low	0		0		0		
Middle	−0.30 (−0.90, 0.30)	0.326	−0.63 (−1.46, 0.21)	0.140	−0.18 (−0.99, 0.62)	0.654	
High	−1.10 (−1.74, −0.46)	0.0008	−1.45 (−2.30, −0.61)	0.0008	−0.58 (−1.39, 0.23)	0.163	
HOMA-IR category							0.090
<2.56	0		0		0		
≥2.56	−0.88 (−1.39, −0.36)	0.0008	−1.07 (−1.77, −0.37)	0.003	−0.53 (−1.27, 0.20)	0.155	

Adjusted for: age subgroups; BMI subgroups; infertility time; AMH; basal FSH; basal LH; basal T; AFC; PCOS phenotypes; triglyceride; protocol. BMI, body mass index; AMH, anti-Müllerian hormone; FSH, follicle-stimulating hormone; LH, luteinizing hormone; T, testosterone; AFC, antral follicle count; PCOS, polycystic ovary syndrome; HOMA-IR, homeostatic model assessment of insulin resistance.

**Table 4:** Multivariate logistic regression analysis of HOMA-IR to pregnancy outcomes.

Variable	Non-Adjusted		Adjust I		Adjust II	
	Crude OR (95% CI)	*p*-Value	Adjusted OR (95% CI)	*p*-Value	Adjusted OR (95% CI)	*p*-Value
biochemical pregnancy						
HOMA-IR	0.99 (0.94, 1.04)	0.626	0.99 (0.93, 1.05)	0.727	1.00 (0.94, 1.06)	0.914
HOMA-IR Tercile						
Low	1		1		1	
Middle	0.90 (0.66, 1.21)	0.475	0.87 (0.63, 1.22)	0.426	0.91 (0.65, 1.27)	0.573
High	0.94 (0.70, 1.27)	0.687	0.95 (0.67, 1.33)	0.749	1.00 (0.70, 1.43)	0.992
HOMA-IR category						
<2.56	1		1			
≥2.56	0.95 (0.74, 1.21)	0.656	0.93 (0.70, 1.22)	0.584	0.97 (0.73, 1.29)	0.829
clinical pregnancy						
HOMA-IR	0.98 (0.93, 1.03)	0.406	0.99 (0.93, 1.05)	0.687	0.99 (0.94, 1.05)	0.829
HOMA-IR Tercile						
Low	1		1		1	
Middle	0.85 (0.63, 1.15)	0.285	0.83 (0.59, 1.15)	0.258	0.85 (0.61, 1.19)	0.343
High	0.89 (0.66, 1.19)	0.432	0.93 (0.66, 1.31)	0.687	0.97 (0.68, 1.38)	0.876
HOMA-IR category						
<2.56	1		1		1	
≥2.56	0.90 (0.70, 1.15)	0.388	0.90 (0.69, 1.19)	0.477	0.94 (0.71, 1.24)	0.658
early miscarriage						
HOMA-IR	1.13 (1.02, 1.26)	0.023	1.08 (0.96, 1.22)	0.204	1.08 (0.96, 1.22)	0.190
HOMA-IR Tercile						
Low	1		1		1	
Middle	1.89 (0.87, 4.10)	0.107	2.07 (0.88, 4.90)	0.097	2.07 (0.88, 4.85)	0.096
High	2.56 (1.23, 5.32)	0.012	2.40 (1.03, 5.58)	0.042	2.33 (1.00, 5.44)	0.049
HOMA-IR category						
<2.56	1		1		1	
≥2.56	2.30 (1.27, 4.16)	0.006	2.21 (1.13, 4.33)	0.020	2.16 (1.11, 4.20)	0.024
miscarriage						
HOMA-IR	1.11 (1.02, 1.22)	0.019	1.04 (0.94, 1.16)	0.407	1.05 (0.94, 1.16)	0.376
HOMA-IR Tercile						
Low	1		1		1	
Middle	1.37 (0.76, 2.47)	0.295	1.08 (0.56, 2.10)	0.820	1.09 (0.56, 2.10)	0.805
High	1.89 (1.08, 3.29)	0.025	1.35 (0.71, 2.56)	0.363	1.32 (0.69, 2.53)	0.401
HOMA-IR category						
<2.56	1		1		1	
≥2.56	1.80 (1.14, 2.86)	0.013	1.38 (0.81, 2.33)	0.234	1.37 (0.81, 2.32)	0.240
live birth						
HOMA-IR	0.95 (0.90, 1.00)	0.045	0.97 (0.91, 1.03)	0.380	0.98 (0.92, 1.04)	0.457
HOMA-IR Tercile						
Low	1		1		1	
Middle	0.85 (0.63, 1.16)	0.304	0.90 (0.64, 1.26)	0.532	0.93 (0.66, 1.31)	0.687
High	0.75 (0.56, 1.03)	0.072	0.87 (0.61, 1.25)	0.455	0.92 (0.64, 1.32)	0.641
HOMA-IR category						
<2.56	1		1		1	
≥2.56	0.77 (0.60, 0.99)	0.044	0.85 (0.64, 1.12)	0.249	0.88 (0.66, 1.18)	0.383

Model I adjusted for: age subgroups; BMI subgroups; AMH; basal FSH; AFC; PCOS phenotypes; protocol; endometrial thickness; embryo stage; embryo transferred number. Model II adjusted for: age; BMI; AMH; basal FSH; AFC; PCOS phenotypes; protocol; endometrial thickness; embryo stage; embryo transferred number. BMI, body mass index; AMH, anti-Müllerian hormone; FSH, follicle-stimulating hormone; AFC, antral follicle count; PCOS, polycystic ovary syndrome; HOMA-IR, homeostatic model assessment of insulin resistance.

## Data Availability

The raw data supporting the conclusions of this article will be made available by the authors, without undue reservation.

## Supplementary Materials

The following supporting information can be downloaded at: https://www.mdpi.com/article/10.3390/jcm12030818/s1, Table S1: Multivariate linear regression analysis of HOMA-IR to ovarian sensitivity index (OSI).
