# Supplementary material for: Impact of Insulin Resistance on Ovarian Sensitivity and Pregnancy Outcomes in Patients with Polycystic Ovary Syndrome Undergoing IVF
Source: J Clin Med. 2023 Jan 19;12(3):818. doi: 10.3390/jcm12030818 (PMC9918062; doi:10.3390/jcm12030818)
Supplement: Supplementary file 1 [file jcm-12-00818-s001.zip › Supplementary Table S1.pdf]

Supplementary Table S1. Multivariate linear regression analysis of HOMA-IR to ovarian sensitivity index (OSI).

Variable	all patients		lean（BMI < 25kg/m^2）		overweight to obese（BMI⩾25kg/m^2）		Test for interaction
	Adjusted β (95% CI)	*P*-value	Adjusted β (95% CI)	*P*-value	Adjusted β (95% CI)	*P*-value	*P*-value
HOMA-IR	-0.21 (-0.32, -0.09)	0.0004	-0.28 (-0.45, -0.11)	0.0016	-0.11 (-0.25, 0.04)	0.146	0.024
HOMA-IR Tercile							0.168
Low	0		0		0		
Middle	-0.09 (-0.68, 0.51)	0.776	-0.45 (-1.28, 0.38)	0.287	0.04 (-0.75, 0.83)	0.920	
High	-0.84 (-1.48, -0.19)	0.011	-1.16 (-2.02, -0.30)	0.008	-0.39 (-1.20, 0.42)	0.342	
HOMA-IR category							0.060
< 2.56	0		0		0		
⩾2.56	-0.62 (-1.13, -0.10)	0.019	-0.72 (-1.43, -0.01)	0.046	-0.42 (-1.14, 0.31)	0.263	

Adjusted for: age; BMI; infertility time; AMH; basal FSH; basal LH; basal T; AFC; PCOS phenotypes; triglyceride; protocol.

BMI, body mass index; AMH, anti-Müllerian hormone; FSH, follicle-stimulating hormone; LH, luteinizing hormone; T, testosterone; AFC, antral follicle count; PCOS, polycystic ovary syndrome; HOMA-IR, homeostatic model assessment of insulin resistance.
